# Supplementary material for: Asynchronous magnetic resonance elastography: Shear wave speed reconstruction using noise correlation of incoherent waves
Source: Magn Reson Med. 2022 Oct 27;89(3):990–1001. doi: 10.1002/mrm.29502 (PMC9792433; doi:10.1002/mrm.29502)
Supplement: Supplementary file 1 — DATA S1 MATLAB code used to generate the numerical simulations of Figure 1 [file MRM-89-990-s002.zip › k-Wave/helpfiles/neper2db.html]

neper2db :: Functions (k-Wave)


# neper2db

Convert nepers to decibels.

## Syntax

```
alpha = neper2db(alpha)
alpha = neper2db(alpha, y)
```

## Inputs

|  |  |
| --- | --- |
| `alpha` | attenuation in Nepers/((rad/s)^y m) |

## Optional Inputs

|  |  |
| --- | --- |
| `y` | power law exponent (default = 1) |

## Outputs

|  |  |
| --- | --- |
| `alpha` | attenuation in dB/(MHz^y cm) |

## See Also

`db2neper`
